# Association between *Fbxl5* gene polymorphisms and partial economic traits in Jinghai Yellow chickens

**DOI:** 10.5194/aab-62-91-2019

**Published:** 2019-03-20

**Authors:** Xuemei Yin, Manman Yuan, Yanjun Duan, Shanshan Zhang, Yulin Wu, Jinyu Wang

**Affiliations:** 1 College of Animal Science and Technology, Yangzhou University, Yangzhou, Jiangsu, China; 2 Institutes of Agricultural Science and Technology Development, Yangzhou University, Jiangsu, China

## Abstract

The *Fbxl5* gene is a member of the *F-BOX* family and plays an important role in maintaining iron homeostasis in cells. In order to reveal the genetic effects of *Fbxl5* gene polymorphisms on body weight (BW) traits and reproductive performance in chickens, *Fbxl5* gene polymorphisms were detected in 363 Jinghai Yellow chickens by PCR-single-strand conformation polymorphism (SSCP) and DNA sequencing methods using three primers. With primer 1, three genotypes (BB, bb, Bb) were detected in the Jinghai Yellow chicken population and two mutations (g. 14257 T > C and g. 14262 T > C) were revealed by gene sequencing. With primer 2, two genotypes (EE, Ee) were detected in the same population and one mutation (g. 19018 G > A), and for primer 3, three genotypes (FF, ff, Ff) and one mutation (g. 19018 G > A) were detected. Four single nucleotide polymorphisms (SNPs) were used to estimate the frequency distributions of the eight haplotypes with PHASE 2.1 software. CTCG was the major haplotype with a frequency of 37.93 %, while the least frequent was TCTA with a frequency of 2.98 %. The BW of haplotype combination H1H8 was higher than that of the other haplotypes and was a dominant combination. In terms of reproductive performance, the age at the first egg of the haplotype combination H9H1 was later than in the other haplotypes, but the mean egg weight at 300 days was relatively optimal. The H1H2 haplotype produced the highest mean egg weight in 300 days, although the total number of eggs in 300 days was smaller in the H2H4 haplotype with the highest at first egg. Therefore, we can consider using the haplotype combination H1H2 for selection. The findings of this study expand the theoretical basis of the use of the *Fbxl5 *gene in the molecular breeding of poultry.

## 1 Introduction

F-box and leucine-rich repeat protein 5 was discovered by Ruiz et al. (2014) using a database search method. It was first identified by Vashisht et al. (2009), using immunoprecipitation, as a subunit of SCF type E3 ubiquitin ligase, which belongs to the F-BOX family and encodes 691 amino acids. As one of the E3 ubiquitin enzymes, Fbxl5 can mediate the transfer of activated ubiquitin molecules from the binding enzyme E2. Different ubiquitinated enzymes target different substrate proteins and determine the specificity of ubiquitination, so that related proteins can be specifically degraded (Darosa et al., 2015). It is also closely involved in the cell cycle, signal transduction, DNA repair, immune response, and DNA regulation (He et al., 2016).

Chen et al. (2014) found that the *Fbxl5* gene plays an oncogenic role in lung cancer. High expression of *Fbxl5* contributes to the occurrence and development of lung cancer. They also found that there was a negative correlation between the protein levels of HSSBI and Fbxl5. The *Fbxl5* gene targets HSSBI to mediate its ubiquitination and subsequent degradation, and to regulate the repair of DNA damage (Chen et al., 2014). *Fbxl5* has been reported to function as a tumor suppressor in the regulation of cancer metastasis in cervical cancer and gastric cancer (Wu et al., 2015, 2016; Xiong et al., 2017). Other studies had shown that the primary role of the *Fbxl5* gene was to mediate the subsequent interpretation of the ubiquitin iron regulatory proteins IRP1 and IRP2 through iron dependence, which plays a crucial role in maintaining iron homeostasis in cells (Ruiz et al., 2014).

**Table 1:** Primer sequences of PCR amplification of the chicken *Fbxl5 *gene.

Primer	Position	Primers sequence(5'–3')	Annealing	Length (bp)
			temp (${}^{\circ }$C)	
P1	exon 3	F:TGAATATGAACAGCTAAACTATGCG	55.7	151
		R:CTTAGTCATTTCAAACACCAAGGA		
P2	exon 4	F:TCAGGTTTTTCAGCCAATGTTGAT	57.3	194
		R:CCTCACCTTGATCTGTCTTCTCG		
P3	exon 5	F:AAGTAACCGGGCACACAACAA	57.2	230
		R:ACTCAACTTCTGTAACTGACAACCT		

**Table 2:** The results of haplotype analysis of the *Fbxl5 *gene.

No.	Number	Haplotype	Frequency (%)
H1	267	CTCG	37.9312
H2	46	CTCA	8.3894
H3	138	CTTG	14.8831
H4	40	CTTA	4.7909
H5	90	TCCG	11.6207
H6	134	TCCA	14.2434
H7	17	TCTG	4.3504
H8	6	TCTA	2.9844

Using specific-locus amplified fragment sequencing (SLAF-seq) and a genome-wide association study (GWAS), we identified 19 single nucleotide polymorphism (SNP) loci that were significantly related to the growth of Jinghai Yellow chickens and mapped them to nine genes. We selected *Fbxl5* as a candidate gene. At present, there are few studies on the *Fbxl5* polymorphism and associated economic traits in chickens. Therefore, the aim of the present study was to investigate the contribution of the *Fbxl5 *gene to a wider range of performance traits in chickens, and, in particular, to confirm whether *Fbxl5* gene polymorphisms exert a significant effect on body weight (BW) and reproductive performance at different ages.

## 2 Materials and methods

### 2.1 Ethics statement

The study protocol was approved by the Animal Care Committee of the Department of Animal Science and Technology of Yangzhou University and conducted in accordance with the guidelines of the Animal Use Committee of the Chinese Ministry of Agriculture. All efforts were made to minimize animal suffering.

### 2.2 Population and sample collection

A total of 363 female Jinghai Yellow chickens were selected randomly from the core group of 12 generations, which is a new breed of high quality broiler bred by Jiangsu Jinghai Poultry Group Co., Ltd, Yangzhou University and Jiangsu Province Animal Husbandry and Veterinary General Station. It has the advantages of identical physique, stable and excellent production and hereditary stability. All birds were hatched on the same day and reared on the ground under the same nutritional and environmental conditions. Nine growth traits, the BW of chickens at day 0(BW0) and weeks 2(BW2), 4(BW4), 6(BW6), 8(BW8), 10(BW10), 12(BW12), 14(BW14), 16(BW16) and five reproductive performance (age at first egg, weight at first egg, initial egg weight, weight in 300 days, mean egg weight in 300 days, total egg number in 300 days), were recorded. Genomic DNA was extracted by the phenolchloroform extraction method, dissolved in Tris-ethylenediaminetetraacetic acid (EDTA) buffer, then quantified by spectrophotometry and then stored at -20 ${}^{\circ }$C until analyzed.

### 2.3 Primer design and variability analysis

According to the gallinaceous *Fbxl5* gene sequences (GenBank Accession NO. NC_006091). Primer 5.0 software was used to design three pairs of primers to amplify fragments that encompassed exon 3, exon 4 and exon 5 of the *Fbxl5* gene (Table 1). Primers were synthesized by Sangon Biotech (Shanghai) CO., Ltd. Polymerase chain reaction (PCR) was performend in a 20 µL reaction volume containing 1 µL of genomic DNA, 0.8 µL of each primer, 7.4 µL of ${\mathrm{dH}}_{2}O$, and 10 µL of 2× Taq Master Mix for polyacrylamide gel electrophoresis (Vazyme Biotech Co., Ltd., Nanjing, China). The PCR thermal profile consisted of pre-denaturation at 94 ${}^{\circ }$C for 5 min, followed by 30 cycles of denaturation at 94 ${}^{\circ }$C for 30 s, annealing at X ${}^{\circ }$C for 30 s, elongation at 72 ${}^{\circ }$C for 35 s and final extension at 72 ${}^{\circ }$C for 10 min, at last 4 ${}^{\circ }$C forever (X is annealing temperature specific for the primer pairs; see Table 1).

For single-strand conformation polymorphism (SSCP) analysis, 2.5 µL of each amplification product was mixed with 7.5 µL of denaturing buffer, heated for 10 min at 98 ${}^{\circ }$C and then cooled on ice for 10 min. Denatured PCR products were subjected to 10 % non-denaturing polyacrylamide (29:1) gel electrophoresis at 250 V for the first 5 min and then 120 V cm${}^{-1}$ for 10 to 12 h. SSCP patterns on the gels were visualized by silver staining. For each genotype, Sangon Biotech sequenced the PCR product of four samples.

### 2.4 Statistical analysis

SHEsis software was used to analyze the genetic linkage disequilibrium at SNP locus and PHSAE 2.1 software was used to analyze the types and frequency of haplotypes. All statistical analysis was based on the General Linear Model (GLM) model of SPSS 19.0. The following statistical model was used:

1D′=DDmax,D=Pij-PiPj,whenD≥0,Dmax=min(PiPj,(1-Pi)(1-Pj)),whenD<0,Dmax=min((1-Pi)Pi,Pj(1-Pj)),2r2=D2Pi(1-Pi)Pj(1-Pj).

$P_{i}$: the frequency of i allele; P${}_{j}$: the frequency of j allele; P${}_{ij}$: the haplotype frequency that carries both i allele and j allele.

3 $$Y_{ij}=\mu +G_{j}+e_{\mathrm{ij}},$$

where Y is the phenotypic value of traits, μ the population mean, $G_{j}$ fixed effects of genotype or diplotype, and e random residual error. Multiple comparisons were performed with least squares means.

## 3 Results

### 3.1 SSCP and sequence analysis 

**Figure 1:**
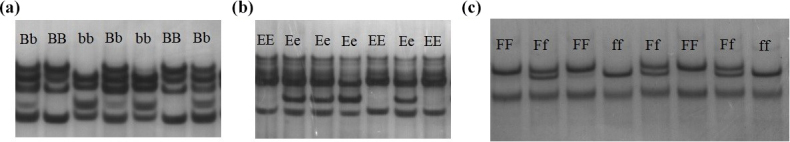
SSCP analysis of PCR amplification with three primers in Jinghai Yellow chicken.

Single-strand conformation polymorphism (SSCP) analysis revealed that the products of the three primer pairs displayed polymorphisms in the *Fbxl5 *gene. Three genotypes were detected in Jinghai Yellow chickens with primer pair 1 (BB, bb, Bb; Fig. 1a), two with primer pair 2 (EE, Ee; Fig. 1b), and three with primer pair 3 (FF, ff, Ff; Fig. 1c).

**Figure 2:**
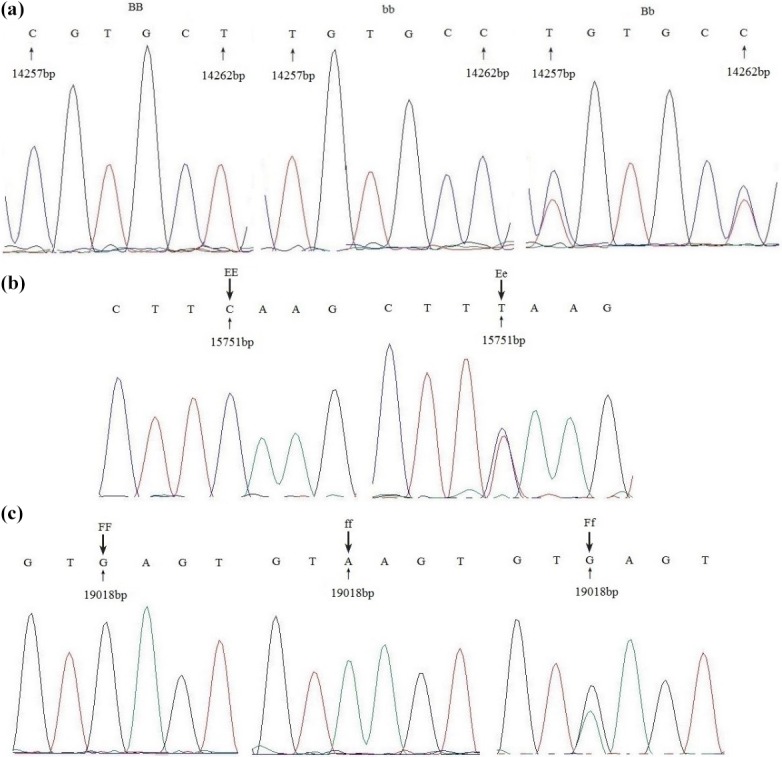
Sequence alignment of three primers with different genotypes in Jinghai Yellow chicken.

The PCR products of different genotypes were cloned and sequenced. Sequencing revealed two nucleotide mutations (g. 14257 T > C and g. 14262 T > C) for primer 1 (Fig. 2a). Sequencing analysis results of the different genotypes for primer 2 are depicted in Fig. 2b; there was one nucleotide mutation (g. 15751 T > C). The results also revealed one nucleotide mutation (g. 19018 G > A) for primer 3 (Fig. 2c). Because of the base substitution, the codon that encodes one amino acid transformed into a codon of another amino acid, translate into different amino acids, which named missense mutation. While with the exist of genetic codon merges, base mutation does not cause amino acid change, which called same-sense mutation. In this study, of all the mutation sites, there was only one (g. 14257 T > C) that caused anamino acid to change, in this case from leucine to proline, which was a missense mutation. Other mutation sites did not result in an amino acid change and so were same-sense mutations.

**Figure 3:**
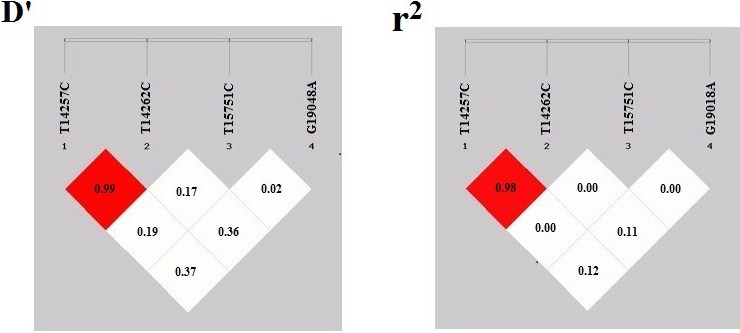
The linkage disequilibrium coefficient $D^{\prime }$ and $r^{2}$ map for the *Fbxl5* gene in Jinghai Yellow chicken.

### 3.2 Linkage disequilibrium analysis of *Fbxl5* mutation sites in different exons

SHEsis online analysis software was used to conduct the linkage disequilibrium analysis of four SNP loci. The results showed that there was a strong linkage disequilibrium between g. 14257 T > C and g. 14262 T > C ($D^{\prime }>0.8$, $r^{2}>0.33$) (Fig. 3). In the image, the red shade represents the depth of the linkage disequilibrium; the deeper the shade of red, the more obvious the linkage imbalance. When the $D^{\prime }$ value was greater than 0.8, the red color was obvious. Values of a coefficient of determination ($r^{2}$) greater than 0.33 denoted a strong linkage disequilibrium and were displayed in red.

### 3.3 Haplotype analysis among mutation sites of *Fbxl5*

Haplotypes and frequencies were analyzed by PHASE 2.1 software. In theory, the number of haplotypes detected in *Fbxl5* should be 16; however, the number of haplotypes obtained using the software was actually 8. The haplotypes with a frequency greater than 1 % were named (Table 2).

### 3.4 Correlation analysis between mutation site haplotype combinations and growth traits 

Correlation analysis between haplotype combinations of four mutation sites of *Fbxl5* and growth traits in Jinghai Yellow chicken by SPSS software. Any haplotype combinations in which the number of individuals was less than three in this population were not discussed. The result showed that the weight of haplotype H2H8 was higher than that of other haplotypes (Table 3). The BW0 of haplotype combinations H1H8 and H3H8 was significantly higher than that of the H1H1, H1H3 and H3H4 combinations (P<0.05). The BW12 of the haplotype combinations H1H3 and H1H4 was significantly higher than that of the H1H1 combination (P<0.05), whereas the BW12 of haplotype combinations H1H8 and H3H8 was extremely significant, higher than those of the H1H1, H1H3, and H1H4 combination (P<0.01). The BW14 of haplotype combinations H1H3, H1H4, H1H8 and H3H8 was significantly higher than that of the H1H1 combination (P<0.05). The BW16 of haplotype combinations H1H8 and H3H8 was significantly higher than that of the H1H1 combination (P<0.05).

**Table 3:** Parameter estimates of *Fbxl5* gene different genotypes on body weight traits (means ± SD).

Weight	Diplotypes					
(g)	H1H1	H1H3	H1H4	H1H8	H3H8	H8H9
BW0	$32.94\pm 0.42^{b}$	$32.06\pm 0.42^{b}$	$32.35\pm 0.76^{b}$	$34.74\pm 0.60^{a}$	$34.53\pm 0.82^{a}$	34.14±0.69
BW2	117.01±1.79	116.41±2.09	115.59±3.45	117.89±2.69	117.56±2.80	117.50±3.73
BW4	244.56±3.93	248.13±4.42	252.64±6.48	256.53±5.99	250.62±6.29	251.79±7.80
BW6	453.01±6.71	460.60±6.88	462.65±9.41	472.89±10.89	462.50±12.06	445.71±12.60
BW8	586.03±8.05	599.25±8.18	605.59±11.24	611.05±17.27	605.94±14.84	605.35±16.55
BW10	763.75±10.47	784.18±9.72	782.65±16.45	797.37±11.94	784.06±10.82	769.69±15.74
BW12	$936.03\pm 12.31^{\mathrm{Bb}}$	$960.42\pm 10.09^{\mathrm{Ba}}$	$961.18\pm 12.07^{\mathrm{Ba}}$	$963.94\pm 16.98^{A}$	$962.50\pm 9.59^{A}$	940.71±11.32
BW14	$1082.93\pm 13.43^{b}$	$1114.48\pm 12.41^{a}$	$1114.71\pm 13.78^{a}$	$1117.47\pm 12.14^{a}$	$1117.03\pm 12.25^{a}$	1106.43±12.33
BW16	$1273.60\pm 14.59^{b}$	1304.46±13.62	1326.76±14.53	$1333.42\pm 13.07^{a}$	$1327.50\pm 15.69^{a}$	1282.14±13.30

Notes: means in the same row with different lowercases indicating significant difference (P<0.05), with different capital letters indicating very significant difference (P<0.01) and no letters indicating no differences (P>0.05).

### 3.5 Correlation analysis between mutation site haplotype combinations and reproductive performance

The relationship between the haplotype combinations of four mutation loci of *Fbxl5* and reproductive performance were analyzed (Table 4). The results showed that the haplotype combination H9H11 was highest in age at the first egg of all the haplotype combinations and was extremely significantly higher than that of the H1H1, H1H3, H1H4, H1H8 and H1H9 combinations (P<0.01). At the same time, the age at the first egg of the haplotype combinations H1H3, H3H8, and H8H8 was significantly higher than that of the H1H1 combination (P<0.05). The weight at the first egg of haplotype combination H1H2 was the heaviest combination, and the weights at the first egg of haplotype combinations H1H2, H1H3, H1H4 and H1H8 were significantly higher than those of the H8H9 and H9H9 combinations (P<0.05). The initial egg weight of the haplotype combination H2H4 was the highest, while that of H4H9 was the lowest. The initial egg weights of the haplotype combinations H1H1, H1H3, H1H4, H1H8, and H2H4 were extremely significantly higher than that of the H4H9 combination (P<0.01), and the initial egg weight of haplotype combination H1H9 was significantly lower than those of the H1H8, H2H4, and H9H10 combinations (P<0.05). The weight at 300 days of haplotype combination H1H2 was the highest, and the weights at 300 days of haplotype combinations H1H2, H1H3, and H1H8 were extremely significantly higher than that of the H8H9 combination (P<0.01). The mean egg weight at 300 days of the haplotype combination H9H11 was the highest, and, with the exception of haplotypes H2H4 and H9H9, the other haplotype combinations were extremely significant (p<0.01) or significantly different from one another (P<0.05). The total egg number at 300 days was highest in haplotype combination H1H4, and the total egg numbers from the haplotype combinations H1H3 and H1H4 were extremely significantly higher than that of the H3H8 combination (P<0.01).

**Table 4:** Parameter estimates of *Fbxl5 *gene different genotypes on reproduction traits (means ± SD).

Traits	Diplotypes													
	H1H1	H1H2	H1H3	H1H4	H1H8	H1H9	H2H4	H3H8	H4H9	H8H8	H8H9	H9H9	H9H10	H9H11
Age at first	143.15±	149.89±	146.89±	147.23±	146.79±	146.50±	144.69±	145.72±	145.30±	148.44±	140.64±	143.62±	140.40±	158.50±
egg/day	$1.14^{\mathrm{Bb}}$	3.02	$1.65^{\mathrm{Ba}}$	$2.64^{B}$	$2.16^{B}$	$4.04^{B}$	3.51	$2.16^{a}$	3.45	$3.19^{a}$	2.47	3.46	3.08	$3.23^{A}$
Weight at first	1615.44±	1706.11±	1668.73±	1687.35±	1672.63±	1642.19±	1671.54±	1642.49±	1573.00±	1665.00±	1546.07±	1566.54±	1615.00±	1640.83±
egg/g	2.65	$5.02^{a}$	$6.88^{a}$	$7.26^{a}$	$6.63^{a}$	4.48	5.42	2.59	8.27	3.14	$7.70^{b}$	$4.17^{b}$	6.00	7.08
Initial	33.38±	30.56±	33.20±	35.29±	36.05±	30.31±	37.30±	31.72±	27.50±	36.11±	31.07±	31.15±	37.00±	32.50±
egg weight/g	$0.96^{A}$	1.30	$0.69^{A}$	$1.63^{A}$	$1.81^{\mathrm{Aa}}$	$2.01^{b}$	$2.63^{\mathrm{Aa}}$	0.86	$1.86^{B}$	2.61	1.90	0.35	$2.90^{a}$	1.11
Weight in	2004.93±	2113.33±	2053.80±	2049.70±	2107.63±	1962.19±	2060.76±	2050.40±	1930.00±	2097.78±	1893.92±	2003.85±	1946.00±	1890.00±
300 days	9.60	$9.98^{A}$	$5.56^{A}$	8.03	$7.05^{A}$	4.56	6.44	5.32	7.58	6.35	7.76	$8.17^{B}$	7.75	8.66
Mean egg weight	51.12±	50.86±	51.66±	51.41±	50.03±	51.51±	49.17±	50.55±	51.25±	52.22±	45.99±	48.26±	52.02±	53.15±
in 300 days	$0.57^{\mathrm{Ab}}$	$2.40^{A}$	$0.56^{\mathrm{Ab}}$	$0.78^{\mathrm{Ab}}$	$1.25^{A}$	$0.86^{\mathrm{Ab}}$	1.77	$0.98^{A}$	$1.22^{\mathrm{Ab}}$	$1.19^{\mathrm{Ab}}$	$3.68^{\mathrm{Ba}}$	2.23	$1.61^{\mathrm{Ab}}$	$1.29^{\mathrm{Ab}}$
Total egg number	104.31±	111.22±	114.34±	117.17±	104.84±	114.68±	104.85±	99.78±	113.80±	101.77±	109.85±	115.69±	116.00±	105.67±
in 300 days	4.33	2.50	$2.62^{A}$	$3.96^{A}$	7.39	2.33	9.22	$7.38^{B}$	6.22	3.16	7.70	5.05	7.70	6.78

Means in the same row with different lowercases indicating significant difference (P<0.05), with different capital letters indicating very significant difference (P<0.01), and no letters indicating no differences (P>0.05).

## 4 Discussion

Iron is an essential element in the life of organisms. Iron in the animal body mainly exists in the form of heme iron and non-heme iron. Salahudeen et al. (2009) and Vashisht et al. (2009) reported that E3 ubiquitin ligase *Fbxl5*, with typical characteristics of F-BOX family proteins, had hemoglobin-like iron-binding regions and was a component of the SCF complex that can respond to intracellular iron levels. It participated in the regulation of iron metabolism through the degradation of the iron regulatory protein IRP2 by the ubiquitin proteasome system (Shen et al., 2012). Jiang et al. (2011) found that heme iron could increase the level of antibody in the blood and improve the disease resistance of laying hens. Gao et al. (2012) found that heme iron had a good growth-promoting effect on weaned piglets, and the blood hemoglobin and IgG concentrations of piglets were increased to some extent.

In this study, we scanned the SNPs in exon 3, exon 4 and exon 5 of the *Fbxl5* gene in Jinghai Yellow chickens. The products amplified by the *Fbxl5* primer pairs 1, 2 and 3 displayed polymorphisms and four SNPs were identified by the sequencing of different PCR fragments, including two (g. 14257 T > C and g. 14262 T > C) located in exon 3 that formed three genotypes (BB, bb, Bb), one (g. 15751 T > C) located in exon 4 that formed two genotypes (EE, Ee), and one (g. 19018 G > A) located in exon 5 that formed three genotypes (FF, ff, Ff). The results indicated that the *Fbxl5* gene had an abundance of polymorphisms in different animal breeds that conform to the comprehensive functions of the *Fbxl5* gene. Linkage disequilibrium usually occurs in natural populations, often expressed in closely linked genes in offspring, so that adjacent SNP alleles are inherited to offspring as a bloc in haplotypes (Johnson et al., 2001). Linkage disequilibrium usually measured in terms of $D^{\prime }$ and $r^{2}$ (Jiang et al., 2004). The results from the current study showed that the two sites involved in the construction of the haplotypes were in strong linkage disequilibrium, $|D^{\prime }|>0.8$, $r^{2}>0.33$, indicating that the loci involved in the construction of haplotypes are closely linked to each other and are largely inherited a bloc (Ardlie et al., 2002; Guryev et al., 2006). Therefore, $r^{2}>0.33$ and $|D^{\prime }|>0.8$ can be used as a measure of strong disequilibrium. The linkage disequilibrium analysis of the four SNP loci detected on three exons of *Fbxl5* gene was carried out; the results showed that there was a strong linkage disequilibrium between g. 14257 T > C and g. 14262 T > C ($D^{\prime }>0.8$, $r^{2}>0.33$) in the core population of Jinghai Yellow chicken, which means that the two markers were closely linked. Therefore, we inferred that the two linked SNP loci may have a certain effect on the same traits.

The results showed that the combined haplotype analysis of multiple loci takes into account the interaction between non-alleles and the linkage disequilibrium prior to the SNP locus, so it has greater statistical potency (Horne et al., 2004). In this study, four SNP loci were found in three exons. There should be 2${}^{n}$ haplotypes in theory, but in fact there were eight effective haplotypes in this population (P>0.01), which may be related to the existence of linkage disequilibrium, the density of the SNP site and the size of sample population. The results showed that only g. 14257 T > C resulted in a change in amino acid encoded, from leucine to proline, among the several SNP sites found in the exon of the *Fbxl5* gene. The other mutations did not cause amino acid change and are, therefore, same-sense mutations. Although the variation in these sites did not cause the change in individual amino acids, the results of haplotype association analysis showed that these polymorphisms caused changes in population growth and reproductive traits, which was consistent with previous studies (Wang et al., 2015).

The results indicated that the *Fbxl5* gene had a significant effect on the growth and development of Jinghai Yellow chickens. Compared with other haplotypes, the body weight of haplotype combination H1H8 was higher than that of the other haplotypes and was a dominant combination, which is the combination that can be considered for selection. The age at which the first age was laid for haplotype combination H9H11 was the highest of all the haplotype combinations, and that for the haplotype combination H9H10 was the lowest of all the haplotype combinations. The weight at the first egg of haplotype combination H1H2 and the initial egg weight of haplotype combination H2H4 was a relatively superior combination. The weight at 300 days of haplotype combination H1H2 and the mean egg weight at 300 days of haplotype combination H9H11 was the highest combination. The total egg number at 300 days of the haplotype combination H1H4 was the relative optimal combination. It can be concluded that the H9H11 combination, which was the later than the other combinations in age at first age, was the best combination in mean egg weight at 300 days. The weight at 300 days of haplotype combination H1H2 was the optimal combination. Whereas the total egg number in 300 days of the haplotype combination with best initial egg weight was smaller than that of other combinations. Therefore, the H1H2 haplotype was regarded as the combination for selection. There was a significant negative correlation between body weight and reproductive performance of poultry. Therefore, in order to improve the potential of rapid and efficient growth and higher reproductive performance at the same time, there had been a number of measures to be taken to limit the amount of feeding (Robinson et al., 1993). Haplotype or haplotype block analysis provides a practical solution to resolving the innate problems of the single-marker analysis, such as noisy, unsatisfied, and obscured localization information (Daly et al., 2001). Both haplotype diversity and the method of SNP selection based on maximum haplotype diversity are always preferred (Huang et al., 2003).

## Data Availability

The datasets used and/or analyzed during the current study are available from the corresponding author on reasonable request.
